# Anti-thyroid drug-induced lupus: a case report and review of the literature

**DOI:** 10.1590/2359-3997000000113

**Published:** 2015-09-18

**Authors:** Xi Mei, Yao Li, Ping Qiu, Ming-Wei Tang, Tian Lan

**Affiliations:** 1 Hospital of Cheng Du Medical College Chengdu Sichuan China The First Affiliated Hospital of Cheng Du Medical College, Chengdu, Sichuan, China

## Abstract

We report a case of drug-induced lupus (DIL) on a Chinese woman caused by methimazole (MMI). This report discusses DIL associated with MMI and briefly reviewed the literature concerning to anti-thyroid DIL.

## INTRODUCTION

Graves’ disease is the most common cause of hyperthyroidism in women of reproductive age. In China, anti-thyroid medication is often the preferred choice for treatment. It is known that, antineutrophil cytoplasmic antibody (ANCA)-associated vasculitis has become increasingly common in patients who have been treated with anti-thyroid medications. However, cases of anti-thyroid DIL are rare. The current diagnostic criteria for DIL as are follows: 1) adequate and sustained exposure to a specific drug, 2) one or more symptoms consistent with lupus, 3) no prior history of lupus, and 4) resolution of symptoms on cessation of the suspected precipitating drug (1).

This report describes a 26-year-old woman with fever, hypersensitivity, and polyarthritis under MMI therapy, and reviewed the findings of a DIL literature search.

## CASE REPORT

A 26-year-old Chinese woman with a 2-day history of pharyngalgia, fever, and 3-day joint pain presented to the First Affiliated Hospital of Cheng Du medical college in Feb 20^th^, 2012. Her medical history included a 1-month history of thyrotoxicosis and medications as MMI (20 mg orally per day). There were systemic multiple migratory joint pain, redness, swelling and generalized muscle pain, especially on the shoulder, neck, and wrist joints.

Physical examination told pharyngeal hyperemia, grade-I thyroid enlargement, joint symptoms (redness, swelling, skin temperature increase) and symptomatic dermatographism. The details were shown on table 1. She considered diagnosis were tonsillitis and thyrotoxicosis. MMI was discontinued and the patient was treated with antibiotics. Although no prior history of drug allergy, she was allergic to cephalosporin, penicillin and levofloxacin at that time. For this reason, we chose azithromycin as an antibiotic agent to treat tonsillitis, and administered a non-steroidal anti-inflammatory drug (NSAID, ibuprofen) as an analgesic treatment. However, body temperature continued to fluctuate between 37 and 39°C. Five days after admission, we proposed treatment with prednisone, but she was transferred to the Chinese People’s Liberation Army General Hospital of Chengdu Military Region. The spectra of antinuclear antibodies (ANA) and autoimmune antibodies shown in table 1 was then examined again and diagnosed as systemic lupus erythematous (SLE). The prescription was prednisone 60 mg orally per day (*q.d.*) with radioactive iodine-131.

**Table 1:** Related index examination after admission

	February 20, 2012 In the First Affiliated Hospital of Cheng Du medical college	February 25, 2012 In the Chinese people’s Liberation Army General Hospital of Chengdu Military Region	April 17, 2012 In the First Affiliated Hospital of Cheng Du medical college	May 27, 2012 In West China Hospital, Sichuan University
ESR	50 mm/h	55 mm/h	70 mm/h	10 mm/h
ANA (< 1:20)	1:320	1:320	—	—
Anti-Sm	—	+	—	—
Anti-ds-DNA	—	—	—	—
ANCA	—	—	—	—
C3, C4 levels	Normal	Normal	Normal	Normal
Albuminuria	Normal	Normal	Normal	Normal
Blood routine	WBC 13.55 × 10^9^/L, N: 80.3%	Normal	WBC 29.84 × 10^9^/L, N: 85.4%	Normal
Temperature	38.5°C	37-39°C	39.2°C	Normal
Antithyroid drugs	MMI, 20 mg, qd	radioactive Iodine-131	PTU, 200 mg, tid	—
Prednisone	—	60 mg, qd	60 mg, qd	—
Arthralgia	+	+	—	—

On May 27^th^, 2012, the patient presented again with a 1-day history of fever, diarrhea and perspiration. The patient had 39.2°C body temperature, 160 beats/min heart rate, and 105/65 mmHg blood pressure. She was sweating heavily accompanying with clammy skin. The patient’s stool tested positive for white blood cells with 3-5 pus cells/hpf upon laboratory examination after admission (Table 1). Her thyroid-stimulating hormone (TSH) level was 0.01 µIU/ml, and triiodothyronine (FT3) and free thyroxine (FT4) levels were slightly elevated. We considered the situation as pre-thyroid crisis or enteritis. She was treated with propylthiouracil (PTU) 200 mg three times a day (*t.i.d.*), propranolol 20 mg every 6 hour (*q.6.h*.), and continued prednisone 60 mg *q.d*. We also adopted other emergency such as levofloxacin (no allergy again) and reducing body temperature. Both temperature and heart rate reduced significantly after 2 days of treatment. Duck salmonella was found in stool culture and ANA spectrum was negative. We then considered a diagnosis of hyperthyroidism and enteritis. She was discharged 1 week later and advised to stop taking PTU. She visited the West China Hospital Sichuan University for follow-up after 40 days of with prednisone 60 mg *q.d*. treatment (Table 1) and ANA spectrum result was negative. Subsequently, her prednisone dosage was gradually reduced and withdrawn 3 years later without discomfort. She was finally diagnosed as DIL and hyperthyroidism.

## DISCUSSION

Since Hoffmann and cols., first reported DIL in 1945, more than 70 related drugs had been reported including chlorpromazine, hydralazine, isoniazid, methyldopa, penicillamine, procainamide, and quinidine, etc. Anti-thyroid drugs MMI and PTU were also associated with DIL (2-6). Zhang and Wang (7) had a study on 229 cases related to DIL from 1975 to 2009 in China and found 42 drugs involved (Table 2). DIL differs from SLE on treatment and prognosis, so it is very important to differentiate them. Generally speaking, DIL case resulted in less renal dysfunction (26.7%) (8), but the pathological changes were analogous to SLE patients. Wang and cols. (9) reported a case of MMI-induced DIL, where focal segmental glomerulosclerosis was observed. Patients with DIL were tested negative for the anti-dsDNA/anti-Sm antibodies, and the complements remained unaffected, whereas in SLE, those antibodies was present and the complements decreased (Table 2) (10-12). In addition, a positive ANA test is required for the diagnosis of DIL (13).

**Table 2:** Comparison the percentage of positive between Drug-induced lupus (DIL) and systemic lupus erythematous (SLE) in laboratory tests

	DIL	SLE
ESR	< 50%	80%
Anemia	< 30%	80%
Leukopenia	< 10%	65%
Thrombocytopenia	< 10%	30%
Albuminuria	< 30%	90%
Hypocomplementemia	< 10%	80%
Rheumatoid factor	30%	50%
ANA	90%	95%
Anti-Smith antibody	0	50%
Anti-dsDNA antibody	0	40%

Both MMI and PTU could be the cause of DIL, and the former’s rate is higher than the latter. The mechanism by which anti-thyroid drugs trigger DIL could be associated with drug-mediated changes to the structure of the DNA-histone complex. The changes lead to the histone not being readily hydrolyzed, causing it to retain immunogenicity or expose new epitopes (14,15). Many drugs are metabolized into cytotoxic metabolites that lead to cell death and abnormal chromatin degradation. This could induce an autoimmune response against histone-DNA complexes in susceptible individuals. Simultaneously, these metabolites may possibly interact with myeloperoxidase to produce ANCA (16). Many patients who are treated with anti-thyroid drugs have abnormal autoimmunity and test negative for ANA and ANCA. Therefore, these drugs may also cause an autoimmune disorder, although the underlying mechanism is still unclear. The patient in this situation should cease medication when the related symptoms turned up. Iodine-131 treatment was recommended under the circumstance.

However, the current diagnosis criterion of SLE firstly recommended by the classification of American association of Rheumatology in 1997 (17) was used to diagnose the case and we considered SLE evidence is still insufficient. Throughout the whole treatment, the case conforms to the diagnosis of DIL. The patient in the case had a MMI medication and typical joint symptoms one month later which was accord with the literature description. Pertinent literature told that DIL was generally observed from 2 weeks to 3.2 years after exposure to a specific drug (3,18). We promptly discontinued MMI therapy and administered non-steroidal anti-inflammatory drugs. The symptoms of DIL usually disappeared several weeks or months after cessation of the drug. Non-steroidal anti-inflammatory drugs were ineffective, so hormones were required until remission.

The patient was misdiagnosed with SLE at the second hospital she visited because of the presence of anti-Sm and arthritis. However, anti-Sm, a symbolized antibody of SLE, was vacant along the whole treatment. Based on this criterion and multiple inspection results of anti-Sm, there was still insufficient evidence to diagnose the patient with DIL (the second positive anti-Sm was an apparent laboratory error.) Subsequently, her prednisone dosage was gradually reduced and withdrawn 3 years later without discomfort. Immunization indicators were normal, and therefore, a diagnosis of DIL was established.

The patient with 1 month history of iodine-131 medication presented to the hospital at second time. PTU was adopted due to pre-thyroid crisis because of symptoms as fever, diarrhea, rapid heart rate (160 beats/min) in admission. Stool/pus cell testing was conducted then, we considered as enteritis whose manifestations resembled pre-thyroid crisis (fever, diarrhea, and dehydration induced rapid heartbeat). Her situation was relieved when anti-inflammatory therapy had been conducted. Pertaining to PTU could be the reason for DIL, anti-thyroid drug was canceled when patient’s situation was under control. Iodine-131 treatment became the better option if the hyperthyroidism symptoms continued in follow-up.

In summary, it is very important to differentiate DIL and SLE. The case with SLE symptoms should be diagnosed carefully when the patient had a history of specific medication. And the immediate cessation of drug was crucial if DIL was considered.
